# Keloid-derived keratinocytes acquire a fibroblast-like appearance and an enhanced invasive capacity in a hypoxic microenvironment *in vitro*

**DOI:** 10.3892/ijmm.2015.2135

**Published:** 2015-03-13

**Authors:** XIAOYANG MA, JIA CHEN, BEI XU, XIAO LONG, HAN QIN, ROBERT CHUNHUA ZHAO, XIAOJUN WANG

**Affiliations:** 1 Department of Plastic and Reconstructive Surgery, Peking Union Medical College Hospital, Chinese Academy of Medical Sciences and Peking Union Medical College, Beijing 100730; 2 Institute of Basic Medical Sciences and School of Basic Medicine, Center of Excellence in Tissue Engineering, Chinese Academy of Medical Sciences and Peking Union Medical College, Beijing 100005, P.R. China

**Keywords:** keloid, keratinocyte, hypoxia, hypoxia-inducible factor-1α, epithelial-to-me senchymal transition, invasion

## Abstract

A keloid scar is an overgrowth of dense fibrous tissue that develops around a wound. These scars are raised scars that spread beyong the margins of the orinigal wound to normal skin by invasion. Keloid tissue consists of both an epithelium and dermal fibroblasts. Recent studies have primarily focused on keloid fibroblasts; however, the precise role of keratinocytes in the invasion process of keloids remains to be identified. Hypoxia is a typical characteristic of keloid scars, as well as other solid tumors. The expression of the transcription factor, hypoxia-inducible factor-1α (HIF-1α), is mainly induced by hypoxia and is known for its ability to induce proliferative and transformative changes in cells; its expression has been shown to correlate with tumor invasion and metastasis. In the present study, we used immunohistochemistry, fluorescence staining and western blot analysis and demonstrated that HIF-1α was highly expressed in both the epithelial layer of keloid tissue specimens and in hypoxia-exposed keratinocytes, which suggested that the keloid keratinocytes underwent epithelial-to-mesenchymal transition (EMT) *in vitro*. The high expression of mesenchymal markers, such as as vimentin and fibronectin was confirmed, as well as the reduced expression of E-cadherin and zonula occludens-1 (ZO-1) during this process by detection at the protein and mRNA level. Moreover, siRNA targeting HIF-1α reversed the changes which had occurred in the morphology of the keratinocytes (cells had acquired a fibroblast-like appearance) and suppressed the invasive ability of the keratinocytes. In conclusion, the present findings demonstrate that the hypoxia/HIF-1α microenvironment provides a favorable environment for keloid-derived keratinocytes to adopt a fibroblast-like appearance through EMT. This transition may be responsible for the enhanced capacity of keloid keratinocytes to invade, allowing the keloids to extend beyond the wound margin.

## Introduction

Keloids are benign fibrous skin tumors which develop due to the overproduction of extracellular matrix (ECM). Keloids occur as a response to dermal injuries and are indicative of an imbalance in wound healing. One of the major characteristics of keloids is the progressive invasion into the adjacent normal skin, exceeding the original wound margin (1–3). The excess deposition of ECM components and the lack of growth regression results in an ischemic and hypoxic microenvironment surrounding the scar tissue (4). This increases the risk of ulceration, which in turn leads to the development of repeated infections, eventually developing into scar carcinomas (5,6). Numerous treatment modalities for keloids are applied alone or in combination; however, there is insufficient evidence to prove their effectiveness.

Previous studies have found that hypoxia exists inside of keloid tissue (7–9). Due to exposure to constant hypoxic conditions, hypoxia-inducible factor-1α (HIF-1α) is highly expressed in keloid tissue and is an important transcriptional regulator which helps cells adapt to the hypoxic microenvironment (8,10). Extensive research has indicated that the stable accumulation of HIF-1α-promotes fibrogenesis in a wide range of tumors through epithelial-to-mesenchymal transition (EMT), which leads to ECM accumulation (11–14).

EMT is a process which leads to the loss of epithelial cell polarity and cell-cell adhesion. Through the process of EMT, epithelial cells acquire migratory and invasive behavior and are thus able to transform into mesenchymal cells. EMT is necessary for several early embryonic developmental processes, including mesoderm formation and neural tube formation (15-17). During the pathological state, EMT is also involved in organ fibrosis, wound healing and in the initiation of tumor metastasis (18–22).

Although the prominence of both hypoxia and the subsequent activation of HIF-1α in the tumor EMT process are known, their functions in regulating keloid pathological processes remain unclear. In the current study, we hypothesized that hypoxia/HIF-1α is a key factor in the transition of keloid keratinocytes into mesenchymal-type cells. This transition enhances the invasive capacity of the keloid-derived fibroblasts. To examine this hypothesis, the expression levels of EMT markers and HIF-1α were determined *in vitro* by reverse transcription- quantitative polymerase chain reaction (RT-qPCR), western blot analysis and fluorescence staining, as well as immunohisto-chemistry. Furthermore, we examined the invasive ability of the keloid keratinocytes under hypoxic conditions using a Transwell co-culture system. Additionally, the involvement of HIF-1α in the transformation of keloid keratinocytes was confirmed by transfecting the cells with siRNA targeting HIF-1α under the same conditions.

## Materials and methods

### Collection of keloid tissue specimens and isolation of keloid keratinocytes

This study was reviewed and approved by the Ethics Committee at the Chinese Academy of Medical Sciences and Peking Union Medical College Hospital (Beijing, China). We collected keloid scar specimens from 30 patients treated at Peking Union Medical College Hospital (Beijing, China). All patients provided written informed consent before participating in this study. All keloid cases were clinically and pathologically proven. Full-thickness skin specimens were harvested from the keloid scar tissues. No patients had previously received any treatment for keloids.

### Cell culture

All skin specimens were incubated overnight at 4°C in dispase II (Sigma-Aldrich, St. Louis, MO, USA) to separated the epidermis from the dermis. Keratinocytes were extracted from the epidermis using 0.25% trypsin (Sigma-Aldrich) at 37°C for 30 min. All cells were filtered through a cell strainer (70 *μ*m; Thermo Fisher Scientific, Inc., Waltham, MA, USA). Primary keratinocytes were incubated in Keratinocyte-SFM (1X) (Life Technologies, Grand Island, NY, USA) supplemented with 10% fetal bovine serum (Invitrogen, San Diego, CA, USA) and 1X penicillin-streptomycin-fungizone (PSF; Life Technologies) for 48 or 72 h at 37°C to allow the cells to adhere to the culture dishes. Non-adherent cells were washed out with phosphate-buffered saline (PBS), and the remaining cells were subcultured or collected for the following analysis at approximately 80–90% confluence to avoid contact inhibition and differentiation. Cells at up to passage 3 were used for analyses.

In addition to the normoxic state, the influence of the hypoxic culture condition on epithelial cells was also determined. When the keratinocytes reached 50% confluence, they were then moved to a hypoxic incubation chamber (New Brunswick Scientific Co., Enfield, CT, USA). The hypoxic condition was maintained by continuous flushing with a mixed gas (1% O_2_, 5% CO_2_ and 94% N_2_). The humidity was maintained, as well as the temperature (37°C).

### Cell morphology

The morphological alterations of the keratinocytes were observed by phase contrast microscopy. The keratinocytes were incubated under hypoxic conditions, and compared to the cells incubated under normoxic culture conditions for 12, 24 and 36 h. The morphological changes were recorded by using phase contrast microscopy and an Olympus inverted microscope (Olympus Corp., Tokyo, Japan).

### Immunohistochemistry

Keloid biopsies were acquired and immediately fixed in 4% formaldehyde for 30 min. The specimens were then embedded in paraffin. Each slide was cut into 5-*μ*m-thick sections. The slides were stained with different primary antibodies for immunohistochemistry, including mouse anti-E-cadherin (ab1416; 1:1,000), rabbit anti-vimentin (Ab92547; 1:1,000) (both from Abcam, Cambridge, UK), mouse anti-fibronectin (sc-8422; 1:400; Santa Cruz Biotechnology, Inc., Santa Cruz, CA, USA) and mouse anti-HIF-1α antibodies (610958; 1:400; BD Biosciences, San Jose, CA, USA). All slides were incubated in biotin-labeled goat anti-rabbit (sc-45101) or goat anti-mouse (sc-395764) serum (1:200; Santa Cruz Biotechnology, Inc.) for 0.5 h, and detection was carried out using the VECTASTAIN Universal ABC-Alkaline Phosphatase kit with ImmPACT NovaRED Peroxidase Substrate (Vector Laboratories, Inc., Burlingame, CA, USA).

### Immunofluorescence staining

The expression of multiple secondary produced mesenchymal proteins, and HIF-1α protein in the epithelial cells was examined by immunofluorescence staining. We isolated primary keratinocytes as described above, then seeded them into dishes at a density of 1×10^5^ cells/well, and waited until they reached 70% confluence in each well under normoxic (21% O_2_) or hypoxic (1% O_2_) conditions. The cells were then fixed with 4% formaldehyde for 10 min. The cells were washed 3 times with PBS and the fixed cells were then treated with EMT-related and HIF-1α primary antibodies (described above) at the appropriate dilution at 4°C overnight, followed by incubation with FITC (sc-65218)- or TRITC (sc-2492)-conjugated secondary antibodies (1:150; Santa Cruz Biotechnology, Inc.) for 1 h at 37°C after extensively rinsing with PBS. Images were captured using an Olympus inverted fluorescence microscope (Olympus Corp.).

### HIF-1α siRNA plasmid construction and cell transfection

The siRNA expression vector for HIF-1α was constructed. To observe the HIF-1α-mediated changes in gene and protein expression under hypoxic conditions, we introduced the HIF-1α siRNA vector, pSilenser 2.1/HIF-si, including a HIF-1α-specific targeting sequence (Table I) and the control scramble siRNA vector, pSilenser, into the keloid keratinocytes. Cell transfection was performed using Lipofectamine 2000 (Life Technologies) according to the manufacturer’s instructions. The transfected cells were cultured for 48 h for further experiments under hypoxic conditions.

### RT-qPCR

We used RT-qPCR to detect the expression of selected genes. Total RNA was extracted from the cultured keratinocytes derived from keloid scars using TRIzol reagent (Invitrogen), followed by treatment with DNase I (Promega Corp., Madison, WI, USA). First-stand cDNA was synthesized with 2 *μ*g total RNA in 30 *μ*l of reaction buffer using a high capacity cDNA synthesis kit (Takara Bio, Inc., Tokyo, Japan) according to the manufacturer’s instructions. Gene expression data were detected using the ABI StepOnePlus system (Applied Biosystems^®^, Life Technologies). The thermal cycling parameters were 95°C for 1 min, followed by 40 cycles of 95°C for 10 sec and 60°C for 40 sec. qPCR was performed to detect the mRNA levels using SYBR-Green I (Takara Bio, Inc.). The expression levels of genes were normalized to β-actin housekeeping gene expression. All RT-qPCR reactions were performed in technical triplicates. The primer sequences used for amplication are listed in Table I.

### Western blot analysis

The protein levels of epithelial and mesenchymal markers in the keratinocytes were quantified by western blot analysis. Total protein was extracted using RIPA lysis buffer (Beyotime, Shanghai, China) which included complete Protease Inhibitor Cocktail (Sigma-Aldrich) and supernatants were collected by centrifugation at 13,000 × g at 4°C for 30 min. The protein concentration was detected using a QuantiPro BCA assay kit (Sigma-Aldrich) followed by separation on a 10% sodium dodecyl sulfate polyacrylamide gel. The proteins were relocated to 0.2 *μ*m polyvinylidene difluoride membranes (Millipore, Billerica, MA, USA). The proteins were blocked with Tris-buffered saline (TBS) with 5% skim milk for 1 h at room temperature. The membranes were then incubated with primary antibodies overnight at 4°C. Mouse anti-E-cadherin (ab1416; 1:1,000; Abcam), rabbit anti-zonula occludens-1 (ZO-1; sc-10804) (1:400; Santa Cruz Biotechnology, Inc.), rabbit anti-vimentin (Ab92547; 1:1,000; Abcam), mouse anti-fibronectin (sc-8422; 1:400; Santa Cruz Biotechnology, Inc.), mouse anti-HIF-1α (610958; 1:400; BD Biosciences) and mouse anti-β-actin (1:4,000; Sigma-Aldrich) were used as primary antibodies. Horseradish peroxidase-labeled goat anti-mouse/rabbit IgG (Beyotime) was then added as the secondary antibody. Signals were visualized using an Immobilon Western Chemiluminescent HRP Substrate (WBKLS0100; Millipore) and image detection was performed using the ImageQuant LAS 4000 mini imaging system (GE Healthcare Bio-Sciences, Pittsburgh, PA, USA). The results were normalized to β-actin (Sigma-Aldrich) where appropriate.

### In vitro invasion assays

The invasiveness of the keloid keratinocytes under normoxic and hypoxic conditions was measured by Transwell co-culture assay. The keratinocytes (2.0×10^5^) were seeded into the top chamber of 24-well plates (pore size, 8 *μ*m; Corning, Inc., Corning, NY, USA) covered with Matrigel (Coating Matrix; Life Technologies) and incubated in full supplemented medium. The lower chambers were contained with chemoattractant (20% serum culture medium). The whole plates were placed into either a normoxic or hypoxic chamber for incubation 12, 24 and 36 h for observation. In addition, the keratinocytes transfected with HIF-1α siRNA, control scramble siRNA and the untransfected keratinocytes were also analyzed in triplicate wells. For those cells that did not go through the membranes, they were wiped out using a cotton swap. Those traversed cells were fixed in methanol and stained in Crystal violet. Phase contrast microscopy was used to record digital images. Three microscopic fields were recorded in each well. At the same time, the elution of Crystal violet was measured at 570 nM spectrophotometer absorbance.

### Statistical analysis

Continuous data are expressed as the means ± standard deviation. For statistical analysis, the Student’s t-test was carried out using SPSS Statistics 19.0 software (SPSS, Inc., Chicago, IL, USA). A value of P<0.05 was considered to indicate a statistically significant difference.

## Results

### Expression of mesenchymal markers and HIF-1α in the epithelial layer of keloid specimens

**Figure 1:**
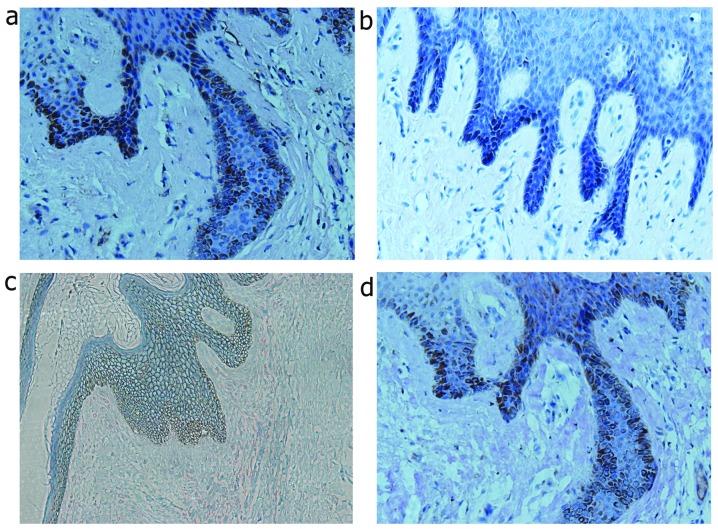
Analysis of epithelial-to-mesenchymal transition (EMT) markers and hypoxia-inducible factor-1α (HIF-1α) expression in keloid scar tissue. Immunohistochemical staining for (a) HIF-1α, (b) vimentin, (c) E-cadherin, and (d) fibronectin in keloid scar tissue specimens.

To demonstrate EMT characteristics in the keloid scars, keloid scar biopsy specimens were examined by histological analysis as shown in Fig. 1. Among these specimens, the epithelial cells clearly underwent EMT, as shown by the abnormal expression of the mesenchymal markers, vimentin and fibronectin, at the basement membrane zone of the epithelial layer, which is the migration zone to the dermis. However, epithelial markers, such as E-cadherin also exhibited a slightly decreased expression around the basement membrane area, which confirmed the occurence of transformation. In addition, HIF-1α was also stained in the same specimens, which coincidentally had a high expression at the same location as the mesenchymal markers (Fig. 1).

### Induction of mesenchymal phenotypic transformation in human keratinocytes under hypoxic conditions

**Figure 2:**
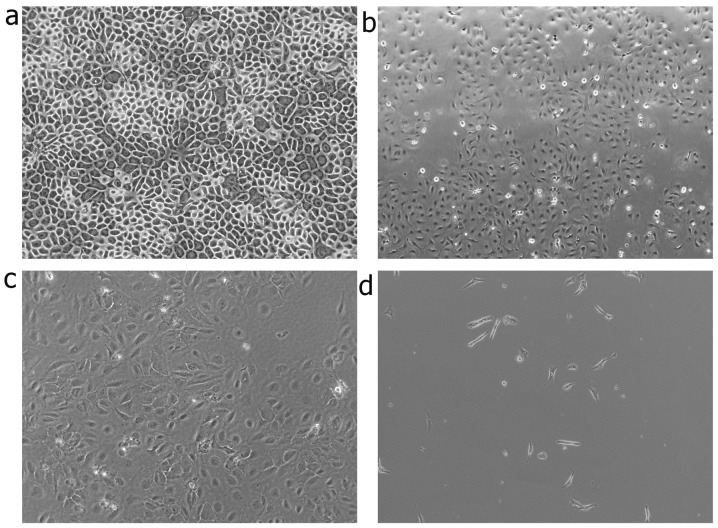
Morphological changes of keloid keratinocytes. Keloid keratinocytes were cultured under normoxic (21% O_2_) and hypoxic conditions (1% O_2_). (a) Keloid keratinocytes under normoxic conditions exhibited an epithelial cell morphology with a typical cobblestone-like growth pattern. (b) Some of the keloid keratinocytes acquired a spindle fibroblast-like shape at 12 h. (c) A greater number of keloid keratinocytes acquired a fusiform fibroblast-like shape at 24 h. (d) After 36 h of epxosure to hypoxia, all cells displayed a significant elongated spindle fibroblast-like morphology (x40 magnification).

Morphometric analysis was performed to further estimate the degree of EMT induction under hypoxic culture conditions. The cells were observed at 12, 24 and 36 h. The keratinocytes cultured under normoxic conditions displayed a characteristic polygonal and cobblestone monolayer morphology. Following culture for 12 h under hypoxic conditions, phenotypic changes, from trivial to remarkable, were observed. Compared to the control cells (cultured under normoxic conditions), the hypoxia-stimulated keratinocytes displayed an elongated, spindle-shaped similar morphology, resembling fibroblasts (Fig. 2).

### Expression of genes in keratinocytes under both hypoxic and normoxic conditions in vitro

**Figure 3:**
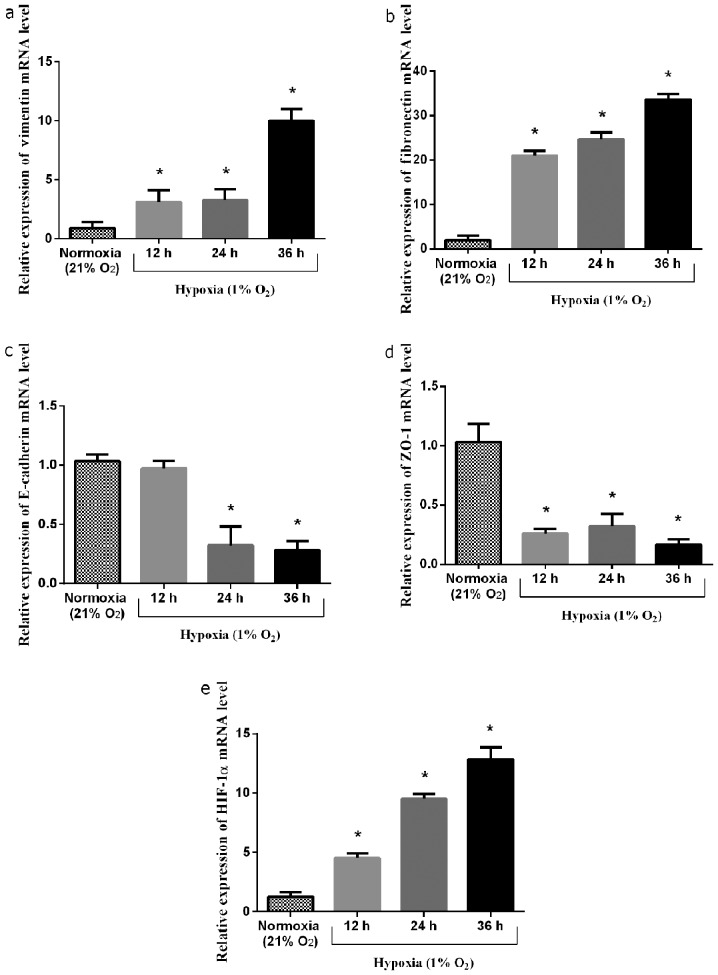
Analysis of alterations in epithelial-to-mesenchymal transition (EMT) marker and hypoxia-inducible factor-1α (HIF-1α) gene expression in keloid keratinocytes. Keloid keratinocytes were cultured under normoxic (21% O_2_) and hypoxic conditions (1% O_2_) for different periods of time (12, 24 and 36 h). The relative mRNA expression of (a) vimentin, (b) fibronectin, (c) E-cadherin, (d) zonula occludens-1 (ZO-1) and (e) HIF-1α in keloid keratinocytes is indicated. *P<0.05 vs. keratinocytes under normoxic conditions (21% O_2_). Bars represent the means ± SD of 3 independent experiments.

The induction of the expression of EMT marker genes was measured under both normoxic and hypoxic conditions. Following incubation for 12, 24 and 36 h in 1% oxygen, the mRNA level of HIF-1α increased by 3.5-, 7.5- and 10-fold, respectively (P<0.05, 0.05 and 0.05, respectively) compared to the controls. Accompanied by the increase in HIF-1α mRNA expression, the mRNA expression of vimentin increased by 3.4-, 3.6- and 11-fold, respectively (P<0.05, 0.05 and 0.05, respectively) compared to the controls. The mRNA expression of fibronectin increased by 10.5-, 12- and 17-fold, respectively (P<0.05, 0.05, and 0.05, respectively) compared to the controls. However, the mRNA expression of ZO-1 was suppressed under the hypoxic conditions by approximately 74.8±1.7% at 12 h, 69.0±3.3% at 24 h and 84.2±2.7% at 36 h compared to the controls (P<0.05, 0.05 and 0.05, respectively). The mRNA expression of E-cadherin in response to the hypoxic conditions decreased by approximately 5.8±1.3% at 12 h, 68.7±5.1% at 24 h and 72.6±2.3% at 36 h compared to the controls (P=0.30, P<0.05 and 0.05, respectively) (Fig. 3).

### Expression of EMT markers in human keratinocytes under hypoxic conditions in vitro

**Figure 4:**
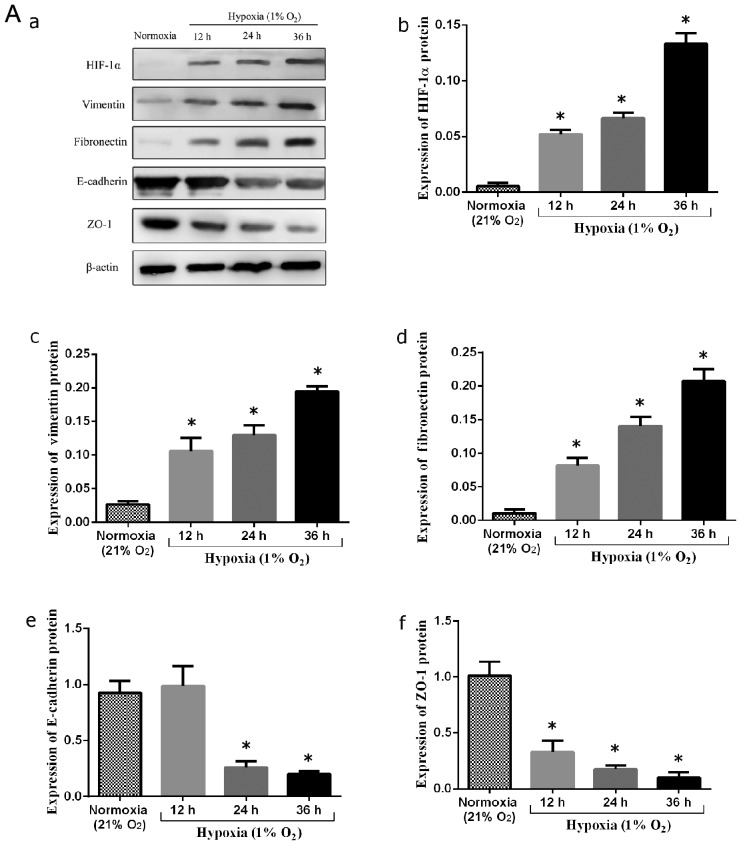

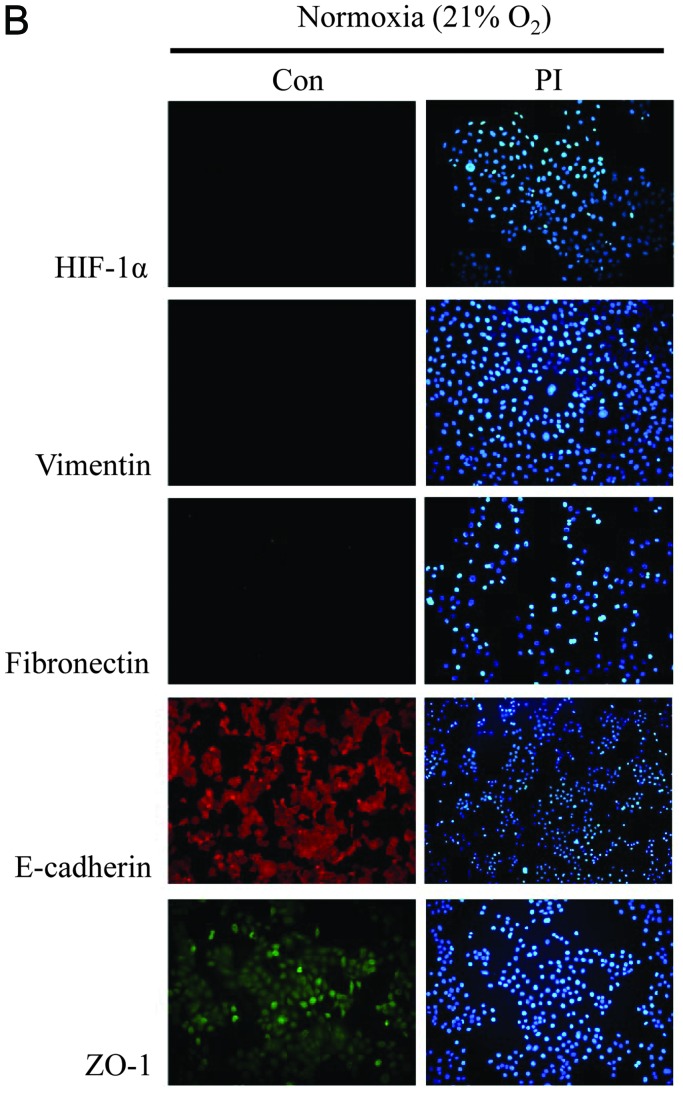

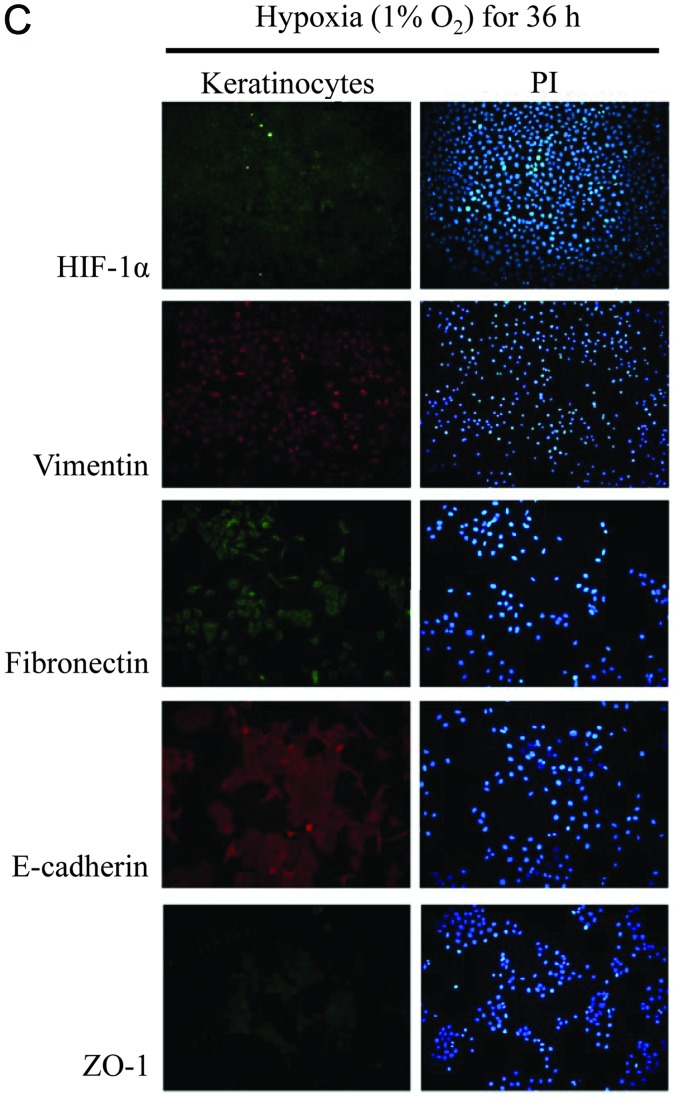
(A) Analysis of alterations in epithelial-to-mesenchymal transition (EMT) marker and hypoxia-inducible factor-1α (HIF-1α) protein expression in keloid keratinocytes. (a) Keloid keratinocytes were cultured under normoxic (21% O_2_) and hypoxic conditions (1% O_2_) for different periods of time (12, 24 and 36 h). The expression of (b) HIF-1α, (c) vimentin, (d) fibronectin, (e) E-cadherin, and (f) zonula occludens-1 (ZO-1) in keloid keratinocytes is indicated. *P<0.05 vs. keratinocytes under normoxic conditions (21% O_2_). Bars represent the means ± SD of 3 independent experiments. (B) Phenotype detection of keloid keratinocytes under normoxic conditions (21% O_2_) by immunofluorescence staining. The expression of HIF-1α and EMT-related markers was not detected in the nucleus or cytoplasm of the keloid keratinocytes after 36 h of culture under normoxic conditions. HIF-1α, vimentin and fibronectin expression was not detected. On the contrary, E-cadherin and ZO-1 were normally expressed and detected under an inverted fluorescence microscope. (C) EMT phenotype detection of keloid keratinocytes under hypoxic conditions (1% O_2_) by immunofluorescence staining. The protein expression of HIF-1α, vimentin and fibronectin was accumulated in the nucleus and cytoplasm of keloid keratinocytes after 36 h of culture under hypoxic conditions. E-cadherin and ZO-1 expression was reduced compared to the cells cultured under normoxic conditions, as observed under an inverted fluorescence microscope.

Due to the association between HIF-1α and EMT markers in keloid scar specimens and hypoxia-induced phenotypic changes in keratinocytes, HIF-1α may potentially provoke or intensify EMT markers during human keloid scar formation. To examine this hypothesis, we first cultured primary keloid-derived keratinocytes in a hypoxic environment (1% O_2_). At 12, 24 and 36 h, the keratinocytes were collected for western blot analysis. In the normoxic control group, vimentin-and fibronectin-positive cells were absent, as shown by western blot analysis. Following culture under hypoxic conditions, the protein epxression of vimentin and fibronectin increased significantly (P<0.05 and 0.05, respectively). By contrast, the epxression of epithelial markers, including E-cadherin and ZO-1 was significantly decreased compared to the normoxic controls (P<0.05 and 0.05, respectively) (Fig. 4A).

To further demonstrate the expression of EMT markers in the cells cultured under normoxic and hypoxic conditions, immunofluorescence staining was performed to evaluate the expression of vimentin, fibronectin, E-cadherin, ZO-1 and HIF-1α. E-cadherin and ZO-1 were strongly manifested in the control group; no expression of mesenchymal markers was observed in the controls (Fig. 4B). However, following exposure to hypoxia, the expression of ZO-1 and E-cadherin was significantly downregulated, and that of mesenchymal markers was increased (Fig. 4C).

### Silencing HIF-1α signaling inhibits the hypoxia-induced mesenchymal transformation of keloid keratinocytes

**Figure 5:**
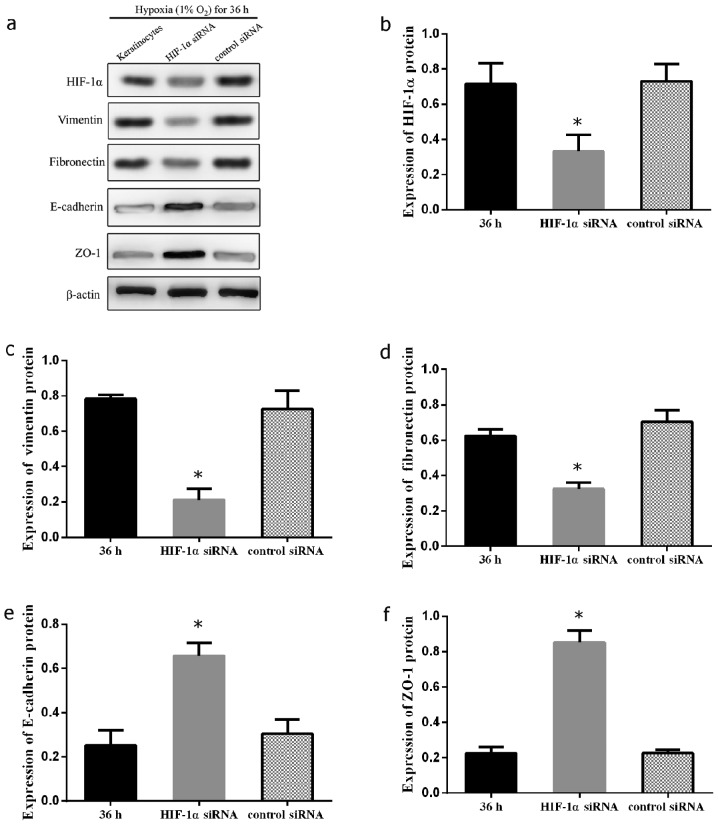
Analysis of epithelial-to-mesenchymal transition (EMT) marker and hypoxia-inducible factor-1α (HIF-1α) protein expression in HIF-1α siRNA-transfected keloid keratinocytes. The expression of HIF-1α was suppressed by HIF-1α siRNA, and was accompanied by the decreased expression of EMT-related proteins. (a) Keloid keratinocytes were transfected with either HIF-1α siRNA or control scramble siRNA prior to exposure to hypoxic incubations for 36 h. They were analyzed and compared to untransfected keratinocytes. The expression of (b) HIF-1α, (c) vimentin, (d) fibronectin, (e) E-cadherin, and (f) zonula occludens-1 (ZO-1) in the keloid keratinocytes is indicated. ^*^P<0.05 vs. 36-h hypoxia-exposed keratinocytes and control scramble-transfected keratinocytes. Bars represent the means ± SD of 3 independent experiments.

**Figure 6:**
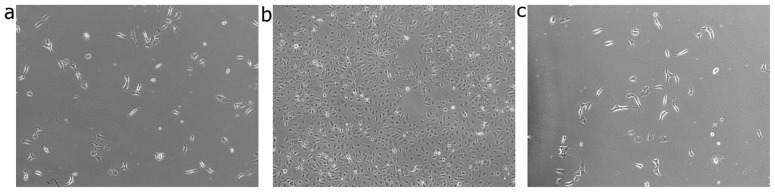
Morphological changes in the transfected keloid keratinocytes. Keloid keratinocytes were transfected with either hypoxia-inducible factor-1α (HIF-1α) siRNA or control scramble siRNA prior to incubation for 36 h under hypoxic conditions. The morphological changes were compared to the untransfected keratinocytes under the same culture conditions. (a) Untransfected keloid keratinocytes at 36 h of exposure to hypoxia exhibited an elongated spindle fibroblast-like morphology. (b) HIF-1α siRNA-transfected keratinocytes persevered their cobblestone shape. (c) Control scramble siRNA keratinocytes still showed a fusiform- like morphology (×40 magnification).

In order to confirm the mechanisms through which HIF-1α participates in hypoxia-mediated EMT in keloid-derived keratinocytes, the HIF-1α gene was knocked down by siRNA in keloid-derived keratinocytes. The expression levels of E-cadherin, ZO-1, fibronectin and vimentin were measured by western blot analysis following the silencing of HIF-1α by introducing HIF-1α siRNA or control scramble siRNA into the keratinocytes under hypoxic (1% O_2_) culture conditions. A significant decrease in the expression of vimentin and fibronectin was observed (P<0.05 and 0.05, respectively). On the contrary, the marked restoration in the expression of E-cadherin and ZO-1 suggested the distinctive importance of HIF-1α signaling in EMT in keloid keratinocytes (P<0.05 and 0.05, respectively) (Fig. 5). The effects of siRNA on keloid keratinocytes under hypoxic conditions were also confirmed by phase contrast microscopy. The cellular morphology of the keratinocytes transfected with HIF-1α siRNA was reversed back to a typical epithelial-like shape compared to the control cells transfected with the scramble siRNA under hypoxic conditions (Fig. 6). This indicated that the mesenchymal changes induced by hypoxia in the keloid keratinocytes were regulated by HIF-1α signaling.

### Invasiveness of normal keratinocytes in vitro under hypoxic culture conditions

**Figure 7:**
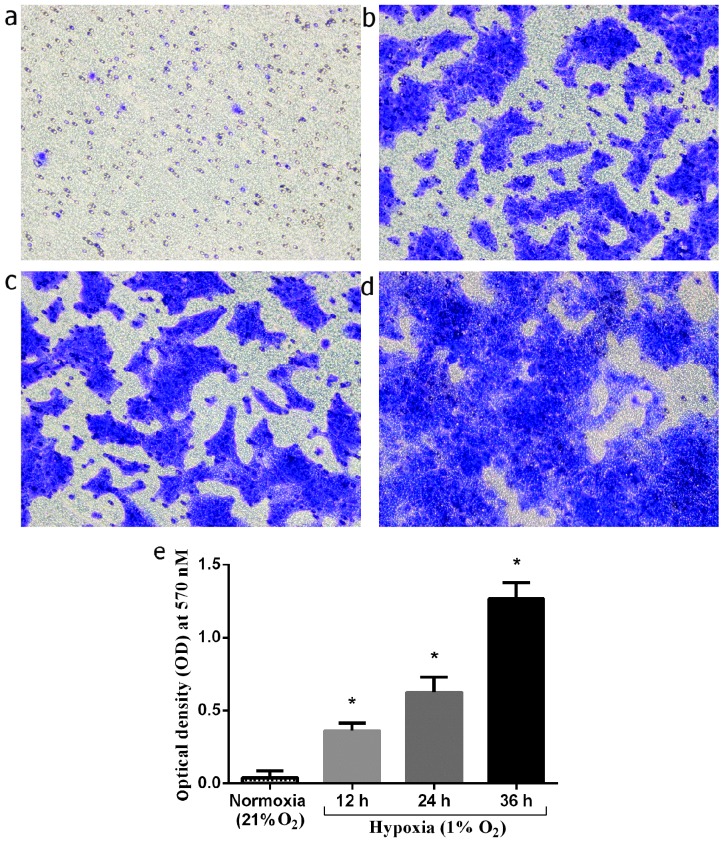
Cell invasiveness investigated by Matrigel co-culture chambers. The invasive cells were fixed and stained with Crystal violet. (a) Invasion of keloid keratinocytes under normoxic conditions (21% O_2_). (b–d) Invasion of keloid keratinocytes cultured under hypoxia conditions (1% O_2_) for 12, 24 and 36 h, respectively. (e) Columns indicate the optical density (OD) of Crystal violet eluted from the invading keloid keratinocytes under normoxic and hypoxic culture conditions (12, 24 and 36 h). ^*^P<0.05 vs. keratinocytes under normoxic conditions (21% O_2_). Bars represent the means ± SD of 3 independent experiments.

**Figure 8:**
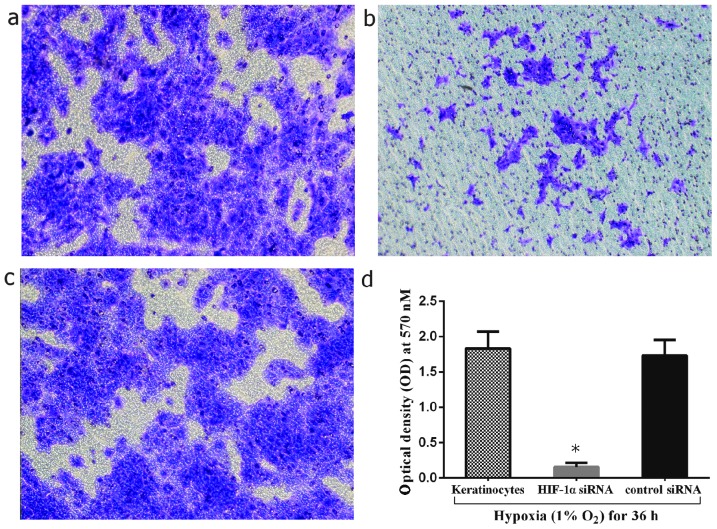
Invasiveness of transfected keratinocytes examined by Matrigel co-culture chambers. Keloid keratinocytes were transfected with either hypoxia-inducible factor-1α (HIF-1α) siRNA or control scramble siRNA prior to incubation under hypoxic conditions for 36 h. The invasive cells were fixed and stained with Crystal violet. (a) Invasion of untransfected keloid keratinocytes under hypoxic conditions (1% O_2_). (b) Invasion of HIF-1α siRNA-transfected keratinocytes and (c) invasion of control scramble siRNA-transfected keratinocytes. (d) Columns indicate the optical density (OD) of Crystal violet eluted from the invading keloid keratinocytes cultured under hypoxic conditions for 36 h. ^*^P<0.05 vs. 36-h hypoxia-exposed keratinocytes and control scramble-transfected keratinocytes. Bars represent the means ± SD of 3 independent experiments.

The stimulating effect of hypoxia on the invasion of keloid-derived keratinocytes was investigated under hypoxic culture conditions. The keratinocytes were cultured under hypoxic conditions (1% O_2_) for 12, 24 and 36 h, and compared to the cells cultured under normoxic conditions. The number of migrated keratinocytes at 12, 24, and 36 h of culture under hypoxic conditions markedly increased by 9-, 15.8- and 32-fold, respectively compared to the normoxic controls (P<0.01, 0.01 and 0.01, respectively) (Fig. 7). In addition, the invasive capacity of the cells was compared between the HIF-1α siRNA-transfected keratinocytes and the hypoxia-exposed keratinocytes. The results revealed a significant decrease in the number of infiltrated cells by 91.3±1.2% in the HIF-1α siRNA-transfected group (P<0.05) (Fig. 8). As a result, hypoxia/HIF-1α may enhance the invasive capacity of keloid keratinocytes.

## Discussion

Keloids are benign fibrous skin tumors characterized by fibroblast proliferation and the excessive accumulation of ECM. Unlike typical hypertrophic scars, keloids can increase in size indefinitely and can spread beyond the original wound margin, which leads to body disfiguration and dysfunction (23,24).

In this study, we detected the expression of HIF-1α, as well as that of epithelial and mesenchymal markers in keloid tissue biopsy speciments. It was observed that HIF-1α was highly expressed in the epithelial layer of the keloid skin specimens, particularly along the basal layer, which was adjacent to the dermis. Furthermore, it should be noted that mesenchymal markers were located in the epidermal basal layer, where E-cadherin was not detected or was expressed at low levels (Fig. 1). Inspired by the evidence *in vivo*, we further confirmed that the epithelial markers, E-cadherin and ZO-1, were downregulated in the primary keloid keratinocytes under hypoxic culture conditions compared to the normoxic control group. In addition, the mesenchymal markers, fibronectin and vimentin, were overexpressed in the hypoxic group in a time-dependent manner. Immunofluorescence staining and western blot analysis also confirmed that HIF-1α siRNA inhibited the EMT process in the keloid keratinocytes under hypoxic conditions. These findings suggest that the inhibition of HIF-1α-mediated EMT transcription factors may be crucial in the repression of keloid development induced by a hypoxic microenvironment.

The majority of relevant studies have focused more on keloid fibroblasts rather than on keloid keratinocytes (25-27). However, certain studies have found that keloid keratinocytes are involved in the keloid pathological process and have a significant impact on the biological characteristics of fibroblasts (28-31). In this study, we confirmed that keloid keratinocytes responded to hypoxic conditions by acquiring a fibroblast-like appearance *in vitro*. This indicates that HIF-1α is involved in the initiation and modulation of the expression of epithelial and mesenchymal markers in keloid keratinocytes. This suggests that keloid keratinocytes may be a potential origin of fibroblasts. To confirm this hypothesis, we also investigated the morphological changes by culturing primary keloid keratinocytes under hypoxic conditions, and our results revealed a distinct epithelial-to-mesenchymal morphological alteration.

Hypoxia is an important characteristic in the tumor tissue microenvironment and is involved in numerous critical pathological developments. It is recognized that the hypoxic microenvironment exists inside of keloids and causes the accumulation of HIF-1α (9). A previous study demonstrated that a high HIF-1α expression in keloids was closely associated with the tumor bioenergetic characteristics and tumor metastasis (3). Several studies have focused on HIF-1α as the key promoter of ECM accumulation (32). However, the role of HIF-1α in keloid formation and growth is still not clearly understood. Our study demonstrated that keloid keratinocytes underwent a transition from an epithelial to a mesenchymal phenotype in response to HIF-1α, with a marked upregulation in the expression of vimentin and fibronectin, and a decrease in E-cadherin and ZO-1 expression. Notably, this HIF-1α EMT signaling was inhibited and reversed by siRNA targeting HIF-1α, suggesting that HIF-1α may be the crucial EMT initiator and mediator in the hypoxia-induced EMT process in keloid scars.

Previously published studies have demonstrated that the hypoxic microenvironment is a favorable condition for tumor cell growth and invasion (33,34). The key characteristic of HIF-1α is the promotion of angiogenesis, which is necessary for cancer tumor growth and invasion. Under the EMT process, keloid keratinocytes lose their epithelial characteristics, and acquire a mesenchymal phenotype. By acquiring a fibroblast-like appearance, the keloids secrete more ECM, and this causes further HIF-1α accumulation, which may exacerbate the keloid malignant growth pattern (9). Our results revealed that the invasive capacity of the keloid keratinocytes cultured under hypoxic conditions (1% O_2_) was higher than that of those cultured under normoxic (21% O_2_) conditions. Notably, the HIF-1α knockdown in the keratinocytes weakened the invasive capacity compared to the cells cultured under hypoxic (1% O_2_) conditions. All the above findings indicate a possible role for hypoxia/HIF-1α in the progression and development of keloids, promoting a suitable microenvironment contributing to invasive spreading.

In conclusion, our study demonstrates that HIF-1α upregulates vimentin and fibronectin expression and downregulates E-cadherin and ZO-1 expression in keloid keratinocytes under hypoxic conditions, which promotes the EMT process and enhances the invasive capacity of the keloid keratinocytes. These findings provide evidence of the influence of hypoxia on EMT in keloids. HIF-1α may thus be an attractive target for genetic and pharmacological modulations of this process. However, the mechanisms underlying the role of HIF-1α in these processes remain to be determined in further research.

## Figures and Tables

**Table I:** Primer sequences.

Gene name	Sense primers	Antisense primers
β-actin	5′-CATCACTATCGGCAATGAGC-3′	5′-GACAGCACTGTGTTGGCATA-3′
HIF-1α	5′-CAAAACACACAGCGAAGC-3′	5′-TCAACCCAGACATATCCACC-3′
HIF-1α-specific target sequence	5′-AAAGAGGTGGATATGTCTGGG-3′	5′-TTTCTCCACCTATACAGACCC-3′
E-cadherin	5′-ATTCTGATTCTGCTGCTCTTG-3′	5′-AGTCCTGGTCCTCTTCTCC-3′
ZO-1	5′-CAACATACAGTGACGCTTCACA-3′	5′-GACGTTTCCCCACTCTGAAAA-3'
Vimentin	5′-AATGACCGCTTCGCCAAC-3′	5′-CCGCATCTCCTCCTCGTAG-3′
Fibronectin	5′-CCCCATTCCAGGACACTTCTG-3′	5′-GCCCACGGTAACAACCTCTT-3′

HIF-1α, hypoxia-inducible factor-1α; ZO-1, zonula occludens-1.
